# The Role of Geography, Diet, and Host Phylogeny on the Gut Microbiome in the Hawaiian Honeycreeper Radiation

**DOI:** 10.1002/ece3.70372

**Published:** 2024-10-16

**Authors:** Maria S. Costantini, Elin Videvall, Jeffrey T. Foster, Matthew C. I. Medeiros, John D. Gillece, Eben H. Paxton, Lisa H. Crampton, Hanna L. Mounce, Alex X. Wang, Robert C. Fleischer, Michael G. Campana, Floyd A. Reed

**Affiliations:** ^1^ Center for Conservation Genomics, Smithsonian's National Zoo and Conservation Biology Institute Smithsonian Institution Washington DC USA; ^2^ School of Life Sciences University of Hawai'i at Mānoa Honolulu Hawaii USA; ^3^ Illinois Natural History Survey, Prairie Research Institute University of Illinois Urbana‐Champaign Champaign Illinois USA; ^4^ Department of Ecology, Evolution and Organismal Biology Brown University Providence Rhode Island USA; ^5^ Institute at Brown for Environment and Society Brown University Providence Rhode Island USA; ^6^ Animal Ecology, Department of Ecology and Genetics Uppsala University Uppsala Sweden; ^7^ Pathogen and Microbiome Institute Northern Arizona University Flagstaff Arizona USA; ^8^ Pacific Biosciences Research Center University of Hawai'i at Mānoa Honolulu Hawai'i USA; ^9^ Pacific Island Ecosystems Research Center U.S. Geological Survey Hawai'i National Park Hawai'i USA; ^10^ Kaua'i Forest Bird Recovery Project, Pacific Cooperative Studies Unit University of Hawai'i at Mānoa Honolulu Hawai'i USA; ^11^ Maui Forest Bird Recovery Project, Pacific Cooperative Studies Unit University of Hawai'i at Mānoa Makawao Hawai'i USA; ^12^ Hawai'i Division of Forestry and Wildlife Hilo Hawai'i USA

**Keywords:** 16S, adaptive radiation, COI, DNA metabarcoding, ITS2, phylosymbiosis

## Abstract

The animal gut microbiome can have a strong influence on the health, fitness, and behavior of its hosts. The composition of the gut microbial community can be influenced by factors such as diet, environment, and evolutionary history (phylosymbiosis). However, the relative influence of these factors is unknown in most bird species. Furthermore, phylosymbiosis studies have largely focused on clades that diverged tens of millions of years ago, and little is known about the degree of gut microbiome divergence in more recent species radiations. This study explores the drivers of microbiome variation across the unique and recent Hawaiian honeycreeper radiation (Fringillidae: Drepanidinae). Fecal samples were collected from 14 extant species spanning the main islands of the Hawaiian archipelago and were sequenced using three metabarcoding markers to characterize the gut microbiome, invertebrate diet, and plant diet of Hawaiian honeycreepers. We then used these metabarcoding data and the honeycreeper host phylogeny to evaluate their relative roles in shaping the gut microbiome. Microbiome variation across birds was highly individualized; however, source island had a small but significant effect on microbiome structure. The microbiomes did not recapitulate the host phylogenetic tree, indicating that evolutionary history does not strongly influence microbiome structure in the honeycreeper clade. These results expand our understanding of the roles of diet, geography, and phylogeny on avian microbiome structure, while also providing important ecological information about the diet and gut microbiota of wild Hawaiian honeycreepers.

## Graphical abstract

We investigated the roles of diet, geography, and genetic relatedness on the composition of the gut microbiome of an adaptive radiation of island birds. Using metabarcoding data, we determined that microbiome variation across birds was highly individualized; however, source island had a small but significant effect on microbiome structure.

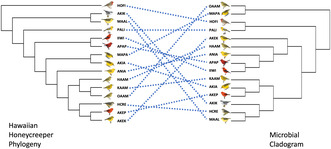

## 1 Introduction

Across vertebrate taxa, host‐associated microbiomes are critical components of host health and fitness (Gilbert, Jansson, and Knight 2014). A single individual can contain billions to trillions of microorganisms, and thus, 50–100 times more genes than those belonging to the host (Hooper and Gordon 2001). These microbes aid in a variety of immune functions (Cerf‐Bensussan and Gaboriau‐Routhiau 2010), digestion (Hanning and Diaz‐Sanchez 2015), nutrient uptake (Douglas 2009), and responses to environmental perturbations (Engel and Moran 2013), and can even influence behavior (Cryan and Dinan 2012). However, microbial composition on or in a host and how those microbes interact with one another varies depending on host taxonomic group (Bodawatta et al. 2022), and even within individuals of the same species.

Various external factors can drive patterns in the diversity and composition of animal microbiomes. For example, sex, age, season, diet (e.g., Bodawatta et al. 2021), geography (e.g., Loo et al. 2019b), and host evolutionary background (e.g., Donohue et al. 2022), can influence which microbes are acquired and incorporated into the gut community. Multiple factors can simultaneously affect gut microbiome composition (e.g., Loo et al. 2019); however, the relative importance of each of these processes in forming an animal's gut microbiome is not yet well understood and seems to differ by taxonomic group and scale (Song et al. 2020).

Several animal clades demonstrate that patterns in host evolutionary history can be tightly linked with patterns in the associated gut microbial communities. This idea, termed “phylosymbiosis,” posits that more distantly related species will have gut microbiomes that are more different from one another (Brucker and Bordenstein 2013). Although evidence of phylosymbiosis seems to be present in some animal clades, it is far from universal. Nonvolant mammals (Song et al. 2020), insects (Van Opstal and Bordenstein 2015), and spiders (Perez‐Lamarque et al. 2022) have shown significant signals of phylosymbiosis. However, other studies have revealed a lack of phylosymbiosis in certain groups (e.g., western chipmunks in North America, Grond et al. 2020). The pattern of phylosymbiosis is not dependent on the process of coevolution between hosts and microbes; it can arise because of shared ecological traits of the hosts, as well as host filtering for certain microbes (Lim and Bordenstein 2020). Thus, even if a phylosymbiotic signal is present, determining the proximate factors responsible may be difficult.

In bird species, most studies investigating the role of phylosymbiosis suggest that host phylogeny does not have a strong influence on the gut microbiome (Kropačkova et al. 2017; Grond et al. 2019; Loo et al. 2019; Fleischer et al. 2020; Song et al. 2020; Trevelline et al. 2020; Bodawatta et al. 2021). However, some that have evaluated birds on a smaller evolutionary scale have demonstrated a weak signal of phylosymbiosis, where phylogeny explains a relatively large part of microbiome variation but still has a relatively small effect size (Baiz et al. 2022; Trevelline et al. 2020). Furthermore, while birds tend to have species‐specific gut microbiomes (Hird et al. 2015), most of the variation in communities is often attributed to intraspecific differences (e.g., Costantini et al. 2021). The mechanisms associated with microbial colonization in birds are expected to differ from other animal clades because of their wide range of dietary niches, high microbial turnover, and, most notably, the physiological gut adaptations for flight (Bodawatta et al. 2021). Birds typically have shorter digestive tracts and gut retention times, suggesting that their microbial communities have high turnover and are more susceptible to environmental changes (Song et al. 2020). This notion is further supported by findings that the microbiomes of flying mammals (e.g., bats) and flighted birds have multiple similarities and that flightless birds have more diverse and stable gut microbiomes like nonflying mammals (Song et al. 2020).

Across avian microbiome studies, diet and habitat are often offered as the most likely factors in explaining gut microbiome assembly processes (Bodawatta et al. 2021). While host phylogeny had a moderate effect in one study of Galápagos finches, diet and habitat correlated more strongly with gut microbiome composition (Loo et al. 2019). Another study found an effect of diet, but not phylosymbiosis in New Guinean passerines (Bodawatta et al. 2021). Yet, in a study on wood warblers, the microbiome more closely matched the phylogeny than diet, but the microbial variation was also significantly affected by geography (Baiz et al. 2022). Diet in avian studies has been characterized in different ways: assigning broad categories like feeding guilds, determining diet from stable isotopes, or using metabarcoding of fecal or gut contents. These methodological differences can influence interpretation of associations between diet and the host‐associated microbiome (Bodawatta et al. 2022). For example, investigating the microbiome of different foraging guilds is useful for broadscale patterns, but is limited in addressing finer‐scale questions. Paired diet‐microbiome data for individual animals can help to tease apart these patterns. Furthermore, it is commonly suggested that the alpha diversity of the diet and the gut microbiome is positively correlated, however, this has rarely been directly tested (e.g., Baiz et al. 2022). Thus, while diet is almost always linked to aspects of the gut microbiome, the relationship between the two is not well understood, especially in wild species that consume natural diets.

Phylosymbiosis studies in birds have largely focused on clades that diverged tens of millions of years in the past (e.g. Hird et al. 2015; Trevelline et al. 2020; Bodawatta et al. 2021), with little known about the influence of evolutionary history on the gut microbiome in more recent species radiations (but see Michel et al. 2018; Loo et al. 2019; Baiz et al. 2022). The Hawaiian honeycreeper radiation (Fringillidae: Carduelinae: Drepanidini) arose from a Eurasian rosefinch (*Carpodacus* spp.) relative that likely arrived from Asia and diverged around the same time as the formation of the oldest main Hawaiian island, Kaua'i, about 5.7 million years ago (Lerner et al. 2011). This avifaunal lineage quickly diversified and filled different ecological niches across the islands, however many species in this radiation have since gone extinct (Paxton et al. 2018). Of the extant honeycreeper species (*n* = 17), only two are considered “Least Concern” by the International Union for the Conservation of Nature (IUCN). All others are of special conservation concern, from threatened to critically endangered (Paxton et al. 2018; see Table 1 for species statuses). From the varying habitats and available food resources came different foraging guilds, often with unique morphological characteristics that are reflected in the radiation of the honeycreeper tree, now providing a unique system to investigate the drivers of microbiome variation. Another unique part of this system is that species of the same dietary specialization have convergently evolved on separate islands in different parts of the honeycreeper phylogeny (e.g., 'Akikiki [*Oreomystis bairdi*] and 'Alawī [*Loxops mana*]; Reding et al. 2009). Because the patterns associated with dietary specialization in Hawaiian honeycreepers are often reflected in phylogenetic patterns, a mechanism for phylosymbiosis may occur in this lineage where it may not in other avian systems. In addition, while some species are single‐island endemics (Paxton et al. 2018), several species occur on multiple islands across the archipelago, which allows for more accurate discrimination between the roles of geography and evolutionary history in gut microbiome composition.

**TABLE 1:** Bird species sampled in the study.

Family	Species	Common name and four‐letter Code	IUCN Conservation status	Foraging guild	Island	Sample size
Fringillidae	*Loxops caeruleirostris*	ʻAkekeʻe (AKEK)	Native, critically endangered	Insectivore	Kauaʻi	8
Fringillidae	*Loxops coccineus*	Hawaiʻi ʻAkepa (AKEP)	Native, endangered	Insectivore	Hawaiʻi	20
Fringillidae	*Hemignathus munroi*	ʻAkiapōlāʻau (AKIA)	Native, endangered	Insectivore	Hawaiʻi	17
Fringillidae	*Oreomystis bairdi*	'Akikiki (AKIK)	Native, critically endangered	Insectivore	Kauaʻi	20
Fringillidae	*Manucerthia mana*	ʻAlawī (HCRE)	Native, endangered	Insectivore	Hawaiʻi	20
Fringillidae	*Magumma parva*	ʻAnianiau (ANIA)	Native, vulnerable	Omnivore	Kauaʻi	27
Fringillidae	*Himatione sanguinea*	ʻApapane (APAP)	Native, least concern	Nectarivore	Hawaiʻi, Kauaʻi, Maui	60 (20 per island)
Fringillidae	*Chlorodrepanis virens*	Hawaiʻi ʻAmakihi (HAAM)	Native, least concern	Omnivore	Hawaiʻi Maui	40 (20 per island)
Fringillidae	*Chlorodrepanis stejnegeri*	Kauaʻi ʻAmakihi (KAAM)	Native, vulnerable	Omnivore	Kauaʻi	17
Fringillidae	*Chlorodrepanis flava*	Oʻahu ʻAmakihi (OAAM)	Native, vulnerable	Omnivore	Oʻahu	5
Fringillidae	*Haemorhous mexicanus*	House Finch (HOFI)	Introduced, least concern	Granivore	Hawaiʻi	8
Fringillidae	*Drepanis coccinea*	ʻIʻiwi (IIWI)	Native, vulnerable	Nectarivore	Hawaiʻi, Kauaʻi, Maui	40 (Hawaiʻi‐28, Kauaʻi‐4, Maui‐8)
Fringillidae	*Pseudonestor xanthophrys*	Kiwikiu (MAPA)	Native, critically endangered	Insectivore	Maui	5
Fringillidae	*Paroreomyza montana*	Maui ʻAlauahio (MAAL)	Native, endangered	Insectivore	Maui	20
Fringillidae	*Loxioides bailleui*	Palila (PALI)	Native, critically endangered	Granivore	Hawaiʻi	9
Zosteropidae	*Zosterops japonicus*	Warbling White‐eye (WAWE)	Introduced, least concern	Omnivore	Hawaiʻi, Kauaʻi, Maui, Oʻahu	40 (10 per island)

A recent study of two endemic insectivorous honeycreepers ('Akeke'e [*Loxops caeruleirostris*] and 'Akikiki) showed that they harbored species‐specific gut microbiomes that are influenced by sampling location and dietary specialization (Costantini et al. 2021). However, a more robust examination of a diversity of honeycreeper species across the radiation with different feeding strategies, and originating from different islands, will better elucidate the eco‐evolutionary dynamics involved in gut microbiome formation. We address this in the present study, by examining honeycreeper gut microbiomes in relation to evolutionary history, diet, and geography. Importantly, DNA from bacteria, invertebrates, and plants were all sequenced from the same samples, and each bird species was represented in the dataset by multiple individuals, often from different localities, to account for intraspecific variation. We used the invertebrate and plant sequences to (1) characterize the herbivorous and insectivorous diets of honeycreepers, (2) examine how dietary composition differs by species, foraging guild, and geography, and (3) assess how diet is associated with the gut microbiome. We further examined (4) the degree to which host phylogeny, diet, and geography influence the structure of honeycreeper gut microbiome communities. The present study provides a unique opportunity to test and discriminate among specific hypotheses to determine the degree and ways that different factors (phylogenetic relatedness, geographic distribution, and diet) act to shape microbiome composition and evolution in Hawaiian honeycreepers, a famously imperiled lineage.

## 2 Materials and Methods

### 2.1 Study Sites and Species

**FIGURE 1:**
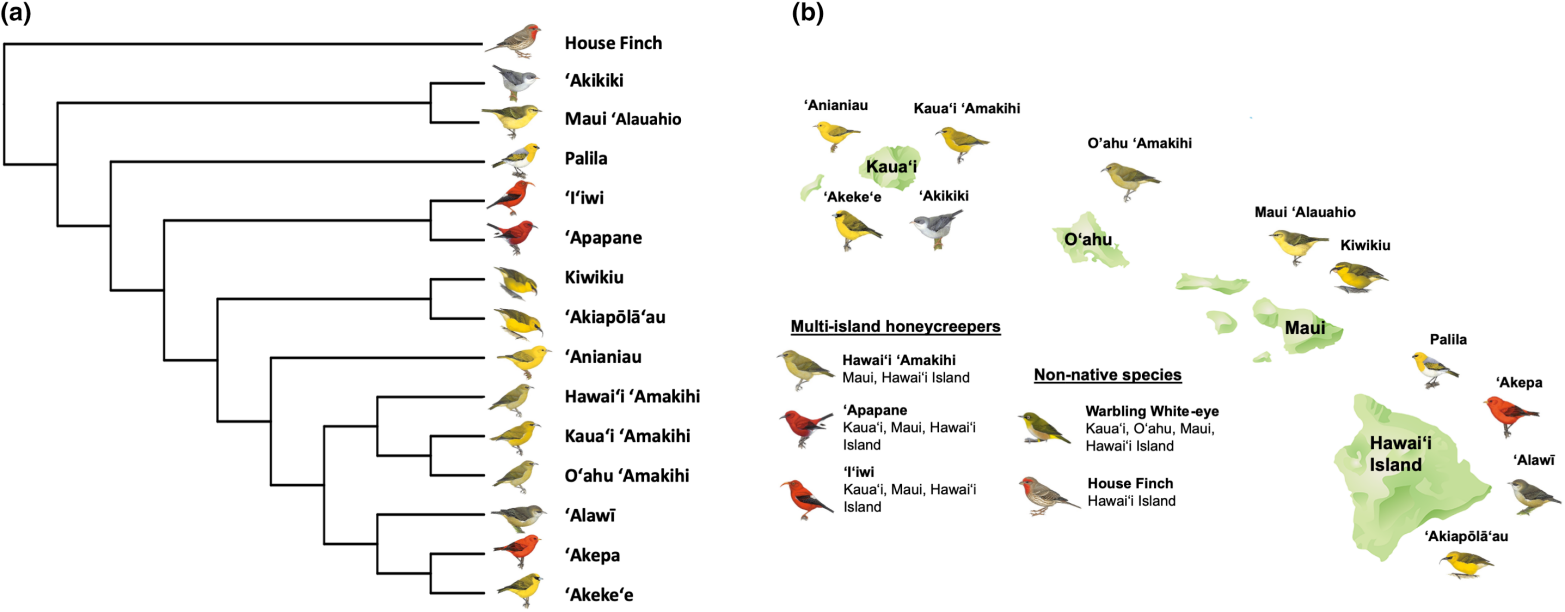
Bird species included in the study. (a) The Hawaiian honeycreeper phylogeny as a cladogram, with House Finch as the outgroup. (b) Map of the Hawaiian islands with species occurrence. Birds around individual islands represent single‐island endemic species. Three honeycreeper species were sampled from multiple islands. Two non‐native species were included: The Warbling White‐eye, which was sampled from multiple islands and the House Finch, which was sampled only on Hawaiʻi Island, and is a close relative to Hawaiian honeycreepers (Image credit: IAN symbols).

Birds were caught in mist‐nets from the islands of Kaua'i, O'ahu, Maui, and Hawai'i from 2012 to 2020 during all seasons. We caught birds at multiple sites on all islands and ranging in elevation from 150 to 1950 m. All extant main‐island Hawaiian honeycreepers (14 of 15; Figure 1) were captured and banded except for the Maui endemic, 'Ākohekohe (*Palmeria dolei*). We were unable to sample the Laysan (*Telespiza cantans*) and Nihoa finches (*Telespiza ultima*), which only occur on uninhabited and remote islands in the Papahānaumokuākea Marine National Monument. All necessary federal and state wildlife research permits and animal care protocols were obtained for this research.

In total, 344 birds from 16 different species were sampled (14 honeycreepers and two introduced songbirds; see Table 1 for species sample sizes). The dataset included species that were single‐island endemics and species that occurred on multiple islands (Figure 1). 'I'iwi (*Drepanis coccinea*) and 'Apapane (*Himatione sanguinea*) were sampled on Kaua'i, Maui, and Hawai'i Island (hereafter referred to as Hawai'i). Hawai'i 'Amakihi (*Chlorodrepanis virens*) were sampled on Hawai'i and Maui. The introduced Warbling White‐eye (*Zosterops japonicus*) was sampled on all islands in this study. Study species also represented all honeycreeper foraging guilds: insectivores, nectarivores, omnivores, and granivores. House Finch (*Haemorhous mexicanus*) samples were included in the study as a non‐native outgroup to the Hawaiian honeycreepers as they belong to the same subfamily (Carduelinae) and they also add a second granivorous species to the study.

### 2.2 Sample Collection

Fecal samples were collected passively from birds while banding. During the banding process, birds were placed into sterilized cloth bird bags for up to 30 min. If a bird deposited a sample, it was collected directly from the bird bag into a microcentrifuge tube filled with 100% ethanol. Bird bags were only used once, and then set aside to be sterilized. When returning from the field, samples were placed in a –20 °C freezer for long‐term storage.

### 2.3 DNA Extraction and Sequencing

DNA was extracted from fecal samples using the Qiagen DNeasy PowerSoil Kit (Qiagen, Germantown, MD, USA) with modifications for diets consisting of arthropod prey (Jusino et al. 2019). Samples were washed with pure water to remove ethanol. A negative control was included in each round of extractions. Extracted DNA was quantified using the Invitrogen Qubit 4 Fluorometer (ThermoFisher Scientific, Waltham, MA, USA). Three different primer pairs were used to sequence the microbiome and dietary components of each sample. The 515F/806R Earth Microbiome Project (EMP) primers were used following the EMP protocol to target the V4 region of the bacterial 16S rRNA gene (hereafter 16S; 390 bp amplicon; Gilbert, Jansson, and Knight 2014). Because the bird species included in the study consume both invertebrate and plant taxa, primers were chosen to target each of these dietary components. The ANML cytochrome oxidase I (COI) primer pair (forward: 5′‐GGTCAACAAATCATAAAGATATTGG‐3′; reverse: 5′‐GGWACTAATCAATTTCCAAATCC‐3′; 180 bp amplicon) was used to specifically target the arthropod components of the diet (following conditions from Jusino et al. 2019). Finally, plant sequences were targeted by including the second internal transcribed spacer (ITS2) primer pair (forward: 5′‐ATGCGATACTTGGTGTGAAT‐3′; reverse 5′‐GACGCTTCTCCAGACTACAAT‐3′; 226 bp amplicon; following conditions from Chen et al. 2010).

Amplicons (polymerase chain reaction [PCR] products) were purified using Mag‐Bind TotalPure NGS beads (Omega Bio‐Tek, Norcross, GA, USA) and index‐tagged to identify the originating bird sample. The final indexed libraries were quantified with the Quant‐iT PicoGreen dsDNA Assay Kit (Invitrogen, Waltham, MA, USA), normalized across sampled individuals, and pooled. The pooled libraries were run on a Bioanalyzer High Sensitivity DNA chip (Agilent, Santa Clara, USA). Samples, plus extraction and PCR negative controls, were sequenced using the Illumina MiSeq platform with the v3 (2 × 300 cycles) reagent kit on three different sequencing runs (each run for a different genetic marker). All library preparation and sequencing were conducted at the Advanced Studies in Genomics, Proteomics, and Bioinformatics (ASGPB) facility at the University of Hawai'i at Mānoa (Honolulu, HI, USA). All samples were processed similarly regardless of target marker. It was not until downstream processing that different steps were taken for each amplicon dataset.

### 2.4 Bioinformatic Analyses

All bioinformatic steps using paired‐end reads were processed within QIIME2 (Bolyen et al. 2019). Raw, demultiplexed amplicon sequences were trimmed of barcoding primers using the Cutadapt plugin (version 4.0; Martin 2011). Reads were quality‐filtered to retain portions of the sequence with Phred quality scores > 30 using the package ʻDADA2’ (version 3.15; Callahan et al. 2016). Reads were then merged, filtered, and chimeras were removed in DADA2. For each primer set, taxonomy was assigned using DADA2's classify‐sklearn() function. Taxonomic identifications of amplicon sequence variants (ASVs) were performed using the SILVA database (version 138) for bacterial 16S, the Barcode of Life database (BOLD), boldsystems.org/, for invertebrate COI following the workflow of O'Rourke et al. (2020), and the DB4Q2 workflow for the plant ITS2 database of Dubois et al. (2022). A feature table containing ASVs, a taxonomy table, and rooted and unrooted phylogenetic sequence trees were exported out of QIIME2 for downstream analyses.

The R package “Decontam” (version 1.14) was used to remove possible contamination from the dataset by removing ASVs that more frequently appear in low‐concentration samples and in negative controls (Davis et al. 2018). Individual samples that contained fewer than 1000 reads and ASVs that occurred fewer than 10 times across samples were removed from the dataset. For bacteria, ASVs matching mitochondria, chloroplast, and Archaea were removed and for COI ASVs that matched fungi were removed. We removed samples from individual birds that were considered “recaptures” from the dataset.

### 2.5 Statistical Analyses

Three different alpha diversity metrics were calculated on each metabarcoding dataset using rarefied reads: richness (observed ASVs) and the Shannon diversity index with the “vegan” R package (version 2.6‐2; Oksanen et al. 2022) and Faith's phylogenetic diversity with the “picante” R package (version 1.8‐2; Kembel et al. 2010). All alpha diversity metrics yielded similar results, so we only report richness. A linear mixed model with Satterthwaite estimates of degrees of freedom was run using the “lme4” R package (version 1.1‐30; Bates et al. 2015) with 16S richness as the response variable and COI and ITS2 richness measures as fixed effects to determine if there was a correlation between diet and microbiome diversity. Bird species and sampling island were included as random effects in the model.

Bacterial and diet community compositions across bird species, foraging guild, and sampling island were calculated with weighted and unweighted UniFrac, Bray–Curtis, and Jaccard for 16S, and Bray–Curtis and Jaccard for COI and ITS2 values using the *distance* function in the “phyloseq” R package (version 1.42.0; McMurdie and Holmes 2013). These distance values were visualized in a Principal Coordinates Analysis (PCoA) ordination plot and compared across bird species, foraging guild, and sampling island. To test for differences between the centroids of groups, a PERMANOVA was run using the *adonis2* function in “vegan.”

Phylosymbiosis was assessed by evaluating the congruence between a microbial dendrogram based on the 16S data in the study and the Hawaiian honeycreeper phylogeny derived from Lerner et al. (2011) (with minor revision based on Campana & Fleischer unpublished data 2020). To create a microbial tree where the number of tips matched the tips of the host phylogeny, samples were first pooled by bird species using the “mean‐ceiling” option of the *qiime feature‐table group* function in QIIME2. This resulted in one data point per bird species. An unweighted pair group method with arithmetic mean (UPGMA) dendrogram was then generated based on unweighted UniFrac distances using the hclust function in R. Relabeling of tip labels to make sure that they matched between trees was done using the “ape” R package (version 5.6‐2; Paradis, Claude, and Strimmer 2004). Tests for congruence were conducted using three different methods; procrustean approach to cophylogeny (PACo; Hutchinson et al. 2017), ParaFit (Legendre, Desdevises, and Bazin 2002), and a Mantel test. The first two methods test for the similarities between trees with statistical significance being assessed by comparison to null models, but PACo uses a procrustean approach (Groussin, Mazel, and Alm 2020). The PACo analysis was conducted using the “paco” R package (version 0.4.2) on the data matrix of branch length distances between hosts, with 1000 permutations and the “quasiswap” null model method, which does not assume that symbionts track the evolutionary history of the host, or vice versa. The ParaFit analysis was performed using the “ape” package and the “cailliez” correction for negative eigenvalues. Lastly, phylosymbiosis was assessed using the correlation‐based method of comparing host patristic distances to 16S unweighted UniFrac distances with a Mantel test in “vegan”.

The relative contributions of host phylogeny, diet, and geography were determined through a multiple regression of matrices (MRM) analysis using the “Ecodist” R package (version 2.0.9; Goslee and Urban 2007). This method is an extension of the partial Mantel test that allows for the testing of multiple predictor distance matrices on a response distance matrix. A series of models was constructed to test the contributions of host phylogenetic dissimilarity, COI (invertebrate) dissimilarity (Jaccard), ITS2 (plant) dissimilarity (Jaccard), and geographic distances on 16S dissimilarity (unweighted UniFrac) using 9999 permutations. Host phylogenetic distances were calculated using the cophenetic function and then turned into a dissimilarity matrix. COI and ITS2 distance matrices were created by converting ASV tables into dissimilarity matrices using the *distance* function in “phyloseq”. The geographic distance matrix was created from latitude and longitude values of the geographic centroid of the islands using the “geosphere” R package (version 1.5‐14; Hijmans 2021). To test if certain diet and microbiome ASVs were associated with different sampling islands or foraging guilds, we used the *multipatt* function from the “indicspecies”R package (version 1.7.14; De Cáceres and Legendre 2009).

## 3 Results

### 3.1 Diet Characterization

**FIGURE 2:**
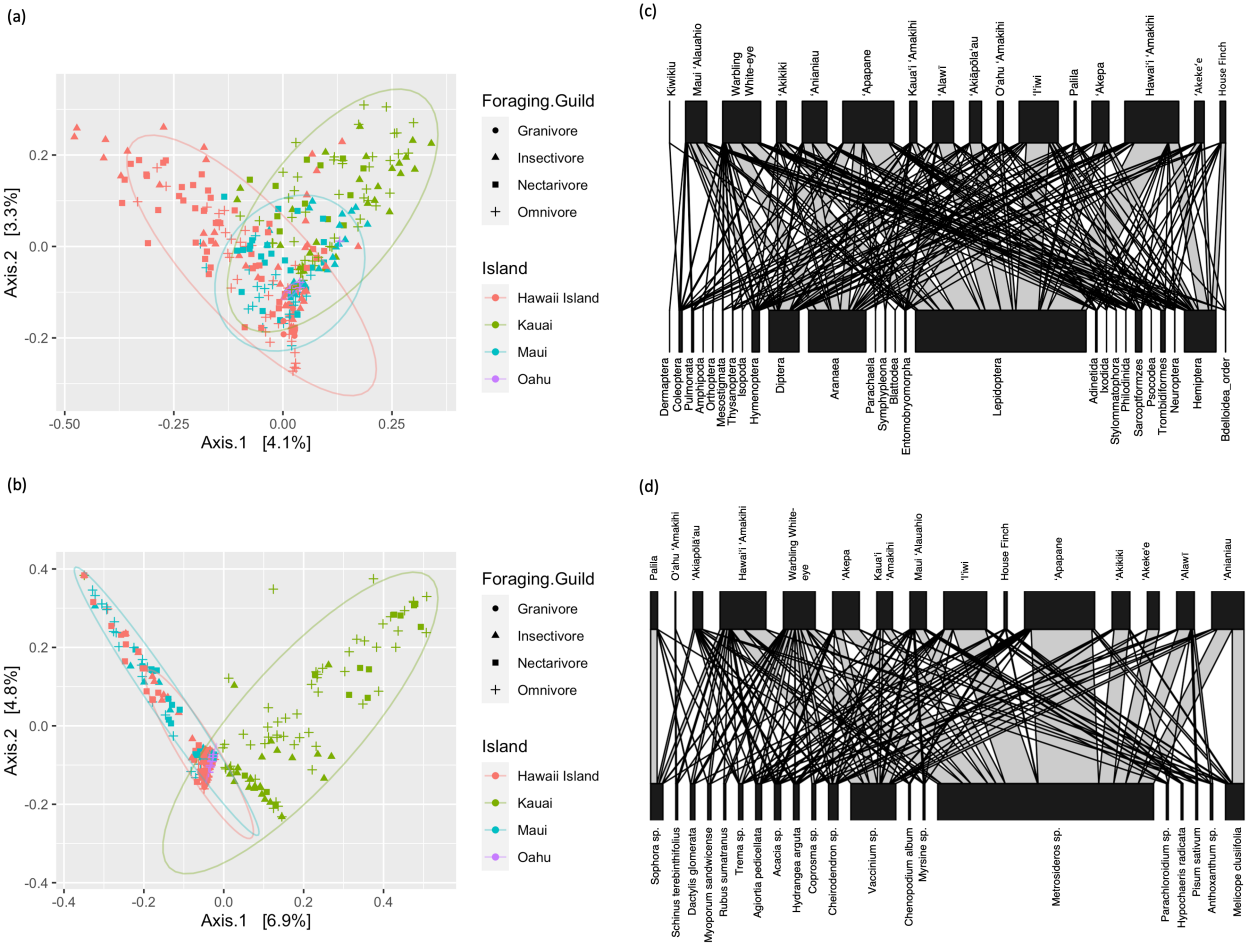
Relationships between birds and components of their diet. Pricinipal coordinate analysis (PCoA) plots showing Jaccard distances of (a) the invertebrate composition and (b) plant composition in bird diets from four Hawaiian Islands. Ellipses represent 95% confidence intervals of islands (colors) and shapes are the four foraging guilds. In (a) most differences are due to islands, with the easternmost island, Hawaiʻi, being the most distant from the westernmost island, Kauaʻi. In (b) Maui, Hawaiʻi, and Oʻahu overlap while Kauaʻi is distinct in space. Bipartite network plots showing the relationships between bird species and components of their diet, displaying (c) invertebrate orders and (d) plant taxa. Horizontal width of bars is a measure of sample size for bird species or frequency of detection for each dietary item. The thickness of the vertical lines indicates the strength of the interaction between bird species and the dietary component.

The COI locus was sequenced to target the invertebrate components of bird diets because all species are expected to consume invertebrate prey to some extent throughout the year. Bioinformatic analyses and filtering yielded 1825 unique COI ASVs; however, less than half were classified beyond Order level (Table S1). The invertebrate community in the diets of birds differed significantly (alpha < 0.05) based on foraging guild (PERMANOVA: *R* ^2^ = 0.02, *p* < 0.001), sampling year (*R* ^2^ = 0.03, *p* < 0.001), and sampling location (*R* ^2^ = 0.23, *p* < 0.001). Sampling location had the greatest *R* ^2^ value and thus accounted for the largest proportion of variation of variables tested. The strong impact of location was also visible in a PCoA of Jaccard distances; Kaua'i and Hawai'i samples separate in ordination space with Maui and O'ahu samples clustering together in the middle (Figure 2). Of the foraging guilds, granivores, in particular, clustered together. 'Akikiki, 'Anianiau, and Maui 'Alauahio had the most diverse invertebrate diets and had significantly higher richness than all other species except for 'Apapane and 'Alawī (based on a Tukey HSD of invertebrate richness; Figure S1).

The Lepidoptera order (i.e., moths and butterflies) was a key component of the diets of all bird species in the study except for the granivorous House Finch, which was almost exclusively associated with Hemiptera (Figure 2) for invertebrate diet items. This order was present in 83% of individuals. The other dominant invertebrate orders in the diet of the bird community included Araneae (65% of individuals), Hemiptera (53% of individuals), and Diptera (43% of individuals).

The ITS2 locus was used to target the plant DNA in fecal samples. Bioinformatic analyses and filtering yielded 1187 unique ITS2 ASVs. Almost all of the reads were classified to at least the Genus level; however, only 20.8% were classified to species (Table S1). The plants in the diet differed significantly based on foraging guild (PERMANOVA: *R* ^2^ = 0.03, *p* < 0.001) and sampling location (*R* ^2^ = 0.28, *p* < 0.001; Figure 2), but not sampling year. In a PCoA of Jaccard distances, Kaua'i separated out in ordination space, with Hawai'i, Maui, and O'ahu falling in the middle (Figure 2). Across the foraging guilds, nectarivores and granivores tended to cluster together, respectively. Warbling white‐eye was the only species that had a significantly more diverse plant diet than any other species ('Akeke'e; based on a Tukey HSD of richness; Figure S1).

The most common plant taxon for birds was the ʻōhia (*Metrosideros* genus), as it was present in 80% of individuals and in every bird species except for O'ahu 'Amakihi (*Chlorodrepanis flava*) and Palila) (*Loxoides bailleui*; Figure 2). The second‐most abundant plant taxon across species was the *Vaccinium* genus; however, this plant was still much less prevalent than the *Metrosideros* genus as it was only in 34% of individuals. As the only native granivore, Palila had a diet almost exclusively of the *Sophora* genus, which includes the native māmane (*Sophora chrysophylla*) of the pea and bean family Fabaceae (Banko et al. 2002). House Finch (introduced) and O'ahu 'Amakihi (native) mostly consumed non‐native plant taxa (e.g., *Schinus terebinthifolius* and *Chenopodium album*).

Within the foraging guilds, nectarivorous, insectivorous, and omnivorous birds mostly consumed the same abundant plant taxa (Figure S2). Granivores consumed the *Sophora* genus and the non‐native grass *Dactylis glomerata* regularly, while the other foraging guilds had no or minimal sequences from these plants. Birds from Hawai'i, Kaua'i, and Maui all consumed similar plant taxa, except only on Kaua'i was there a strong relationship with the native plants alani (*Melicope clusiifolia*), 'olapa and/or lapalapa (*Cheirodendron* spp.), and kanawao (*Hydrangea arguta*). These plant species were a minimal part of bird diets on the other islands despite their presence (Figure S2). Of the 15 most abundant plant taxa in the diet, only O'ahu birds consumed the non‐native *Trema* spp., an indication of the degradation of Oʻahu habitat compared to the other islands.

### 3.2 Avian Gut Microbiomes

The gut microbial communities of all studied species were successfully characterized. The sequencing yielded 9,783,008 reads that resulted in 24,333 unique bacterial ASVs. After filtering for apparent contamination, low yield, and nontarget taxa 20,875 ASVs remained. Of these ASVs, neither read depth or sample size had effects on data comparisons between groups. Of these ASVs, 94% were classified to at least the Family level and only 45.8% were classified to Species (Table S1). The three most abundant bacterial phyla in the gut microbiomes of the entire avian community were Proteobacteria, Firmicutes, and Actinobacteria, with average relative abundances across samples of 37%, 29%, and 26% respectively (Figure S3). Patterns between foraging guilds of birds were largely consistent, aside from a few minor differences. For example, the granivorous species were relatively enriched for Actinobacteria compared to the other guilds, while the insectivorous species tended to have a higher relative abundance of Proteobacteria (Figure S3). The two nectivorous species both had rare phyla, such as Campilobacterota and Bdellovibrionota, in their gut microbiomes that were largely absent in other guilds. Maui ʻAlauahio had the most distinct gut microbiome from all other bird species in terms of the relative abundance of bacterial phyla as it was almost entirely dominated by Firmicutes (Figure S3).

**FIGURE 3:**
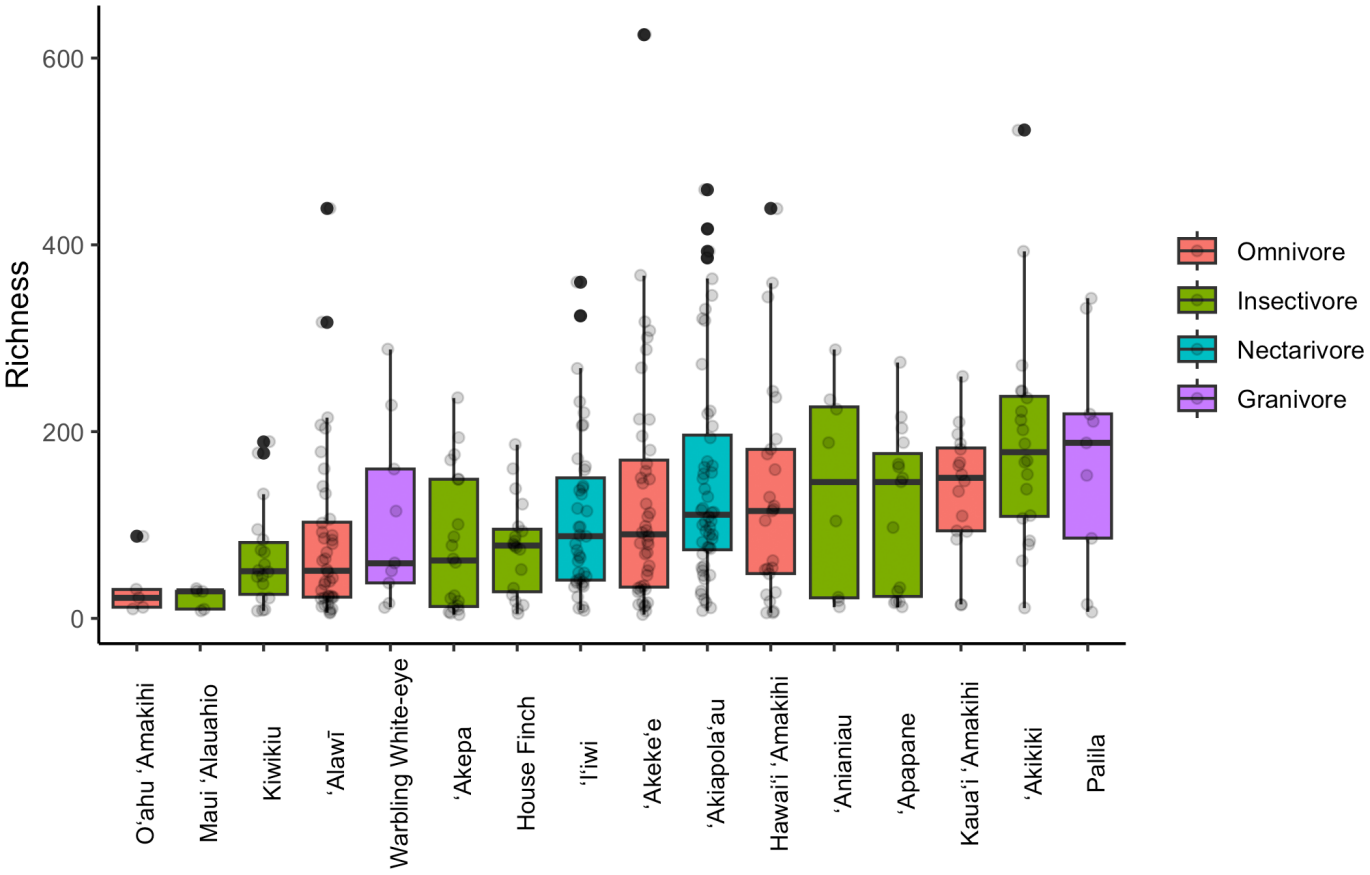
Observed richness boxplot of bacterial amplicon sequence variants (ASVs) by bird species and foraging guild. There is no difference in alpha diversity between foraging guilds. ʻAkikiki is the only species that had a slightly more diverse microbiome compared to Oʻahu ʻAmakihi, Maui ʻAlauahio, ʻAlawī, Warbling White‐eye, ʻAkepa, and Kiwikiu based on Tukey's HSD (*p* < 0.01). The *x*‐axis is ordered by mean richness. Boxplots indicate the median value (bold line in box, 50% quantile (box), and 25% quantiles (whiskers), with outliers denoted by dots.

The insectivorous honeycreeper, ʻAkikiki, had the most diverse gut microbiome of all species, and its richness significantly higher than O'ahu 'Amakihi, Maui 'Alauahio, 'Alawī, Warbling White‐eye, 'Akepa (*Loxops coccineus*), and Kiwikiu (*Pseudonestor xanthophrys*) based on Tukey's HSD on bacterial richness (Figure 3).

### 3.3 Diet and Microbiome Diversity Correlation

**FIGURE 4:**
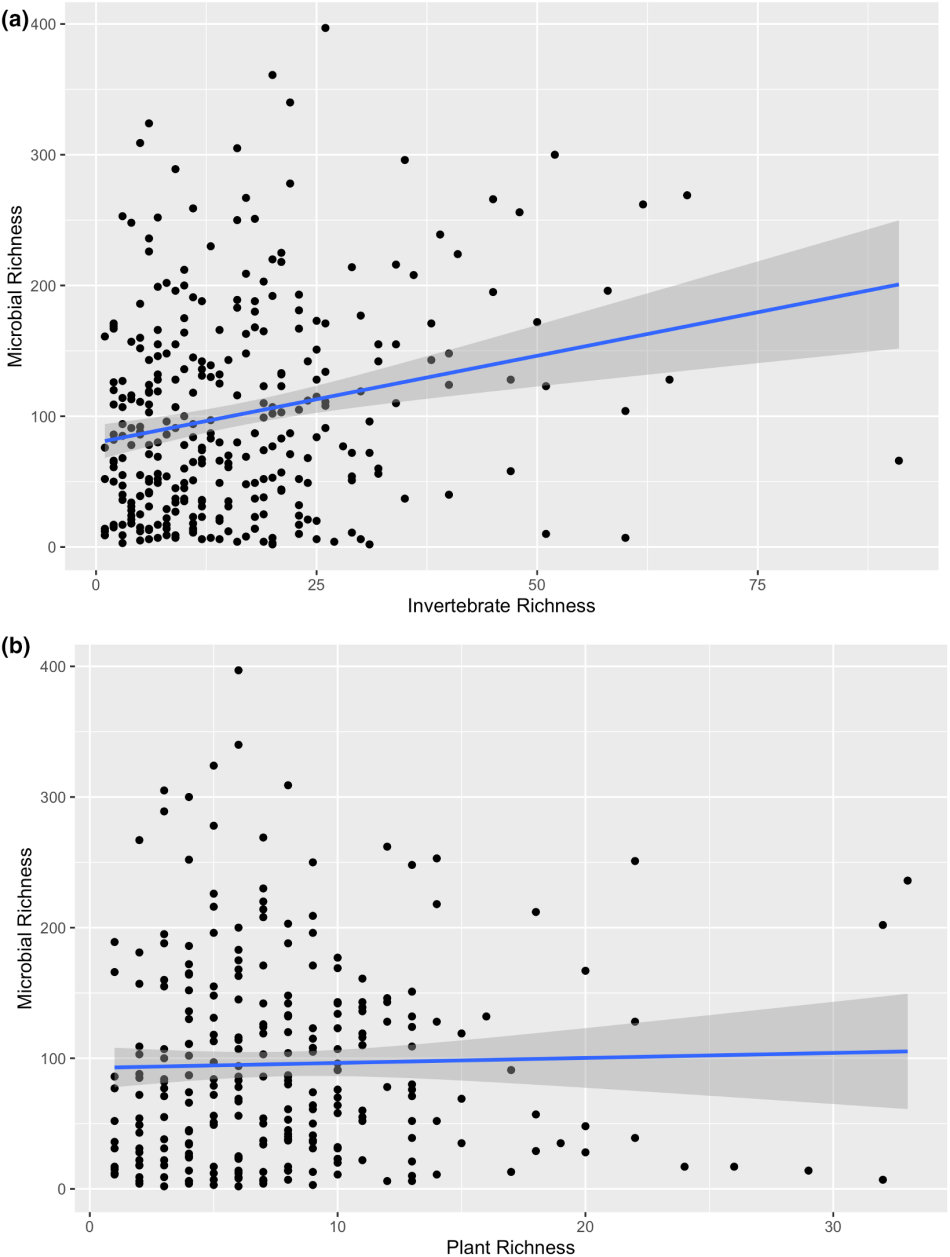
Regression line (with 95% confidence intervals in dark gray) between observed richness of the gut microbiome and observed richness of the (a) invertebrates and (b) plants in bird diets. The trend line in the invertebrate diet linear regression shows a slight positive (R^2^ = 0.05) and significant, correlation (Linear mixed model, *p* < 0.01), while there is no trend in the plant diet linear regression.

Diet diversity is often suggested to positively correlate with microbiome diversity (Heiman and Greenway 2016). To test the hypothesis, a linear mixed model was run with 16S richness as the response variable, COI richness and ITS2 richness as fixed effects, and species and sampling island as random effects. Microbiome richness significantly increased as COI richness increased (*F* = 19.48, *p* < 0.01) but there was no relationship with ITS2 diversity (*F* = 0.25, *p* = 0.80; Figure 4).

### 3.4 Gut Microbiome Composition and Topological Analysis

**FIGURE 5:**
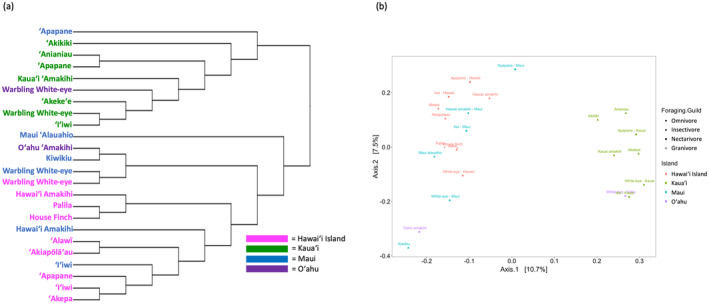
Microbial clustering based on unweighted UniFrac distances. Bird species that occur on multiple islands are shown separately to evaluate their microbiome similarity. In both the microbial dendrogram (a) and the PCoA (b) Kauaʻi samples show more differences from the other islands.

Most of the variation in gut microbiomes was unexplained; however, they tended to cluster together with species from the same sampling island (Figure 5; Figure S4). Most of the diversity metrics yielded similar results. Bray–Curtis was the only one that strayed from this pattern and seemed to follow a slightly more phylogenetic‐based pattern (Figure S4). A PERMANOVA analysis indicated that foraging guild (*R* ^2^ = 0.015, *p* < 0.01) explained a small but significant amount of variation; sampling location (*R* ^2^ = 0.18, *p* < 0.01) explained the most variation, and sampling year was not a significant contributor to beta diversity.

**FIGURE 6:**
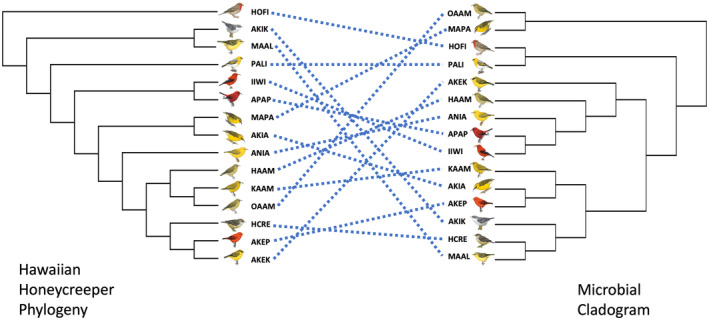
Tanglegram of the Hawaiian honeycreeper phylogeny and each species' respective microbiome based on unweighted UniFrac values. The dashed lines crossing in the tanglegram illustrate the lack of phylosymbiosis between Hawaiian honeycreeper species and their gut microbiomes. Refer to Table 1 for definitions of common name codes.

To test whether phylosymbiosis could explain some of the patterns observed in the gut microbiome of honeycreepers, we used three different analyses. A matrix of unweighted UniFrac distances of the gut microbiome was tested against the patristic distance of the host phylogeny using the correlation‐based Mantel test. This test showed no evidence for phylosymbiosis (Mantel *r* = 0.225, *p* = 0.13). Next, a tanglegram was used to compare a dendrogram from the unweighted UniFrac distances of the gut microbiome to the bird phylogenetic tree by PACo and ParaFit analyses (Figure 6). Neither test found congruence between the microbial dendrogram and the host tree (PACo: *m* ^2^_XY_ = 1.71, *p* = 0.27, nperm = 1000; ParaFit: paraglobal = 5.2 × 10^−2^, *p* = 0.39). Repeating the same analyses using Bray–Curtis values yielded similar results for ParaFit and Mantel, but the PACo test became significant (PACo: *m* ^2^_XY_ = 1.93, *p* = 0.018, nperm = 1000).

### 3.5 Relative Contributions of Phylogeny, Diet, and Geography

To quantify the relative contribution of host phylogeny, plant diet, invertebrate diet, and geographic sampling location on the gut microbiome composition of Hawaiian honeycreepers, each dataset was converted to a distance matrix to run in a series of MRM models. Only the models that included geography as a predictor variable were significant (see Table S2 for statistics). In all models that included geography, geographic distance had a small yet statistically significant positive association with gut microbiome dissimilarity (see Figure S5 for an example of geographic distance versus microbiome dissimilarity in ʻApapane). Host phylogeny also had a significant contribution, but only when geography was also included. The invertebrate and plant components of the diet were not significant drivers of microbiome variation in any model.

To better understand what is driving the variation we see in our models, we ran an indicator species analysis on the bacteria, invertebrate, and plant ASVs compared to sampling island and foraging guild. The island of Oʻahu and the granivores guild had the highest number of correlated ASVs for each marker (Tables S3 and S4; see Table S5 for a list of the 10 most correlated bacterial ASVs for each guild). Both groups are the most distinct (i.e., Oʻahu has the most distinct plant community of primarily non‐native species and the granivore feeding style is rare in Hawaiian honeycreepers) compared to the other groups.

## 4 Discussion

Through metabarcoding, we explored the relative contributions of environmental and evolutionary influences on the structure of the gut microbiome composition throughout the extant Hawaiian honeycreeper radiation. We considered the composition of both the invertebrate and plant components of avian diets and how diet diversity correlated with gut microbiome diversity. We found that most of the variation within the microbiome dataset was unexplained, but there were significant effects of bird species and sampling island. When assessing the degree to which phylogeny, geography, plant diet, and invertebrate diet contribute to gut microbiome dissimilarity, geographic distance was the only variable that was consistently significant regardless of what other variables were included in the model. Host phylogeny was only a significant contributor when geography was also included in the model. Both invertebrate and plant diet components did not contribute significantly to gut microbiome variation.

### 4.1 Diet Characterization

The birds in this study belong to four different foraging guilds across the Hawaiian honeycreeper lineage: insectivore, granivore, nectarivore, and omnivore. However, all are expected to consume invertebrate prey to some extent, especially during breeding and molting (Pratt 2005). The most dominant arthropod order in the diets of all birds was Lepidoptera. The only exception was arthropods in the diet of the introduced granivore, the House Finch, in which the invertebrate component of the diet consisted almost exclusively of Hemiptera. Other arthropod orders such as Araneae, Diptera, and Hemiptera were also present in high abundance across most species, which complements the results of previous invertebrate‐based diet studies of Hawaiian forest birds (Banko et al. 2015; Costantini 2022; Peck et al. 2015). More detailed analyses of Hawaiian bird diets are ongoing to assess interspecific differences as well as spatial and temporal variation.

We also investigated the plant component of the birds' diet. Plant DNA in fecal samples could theoretically represent either consumed plant matter (e.g., seeds, fruit, or nectar) or plant material inside the guts of arthropods eaten by birds. Regardless of the route into the bird's gastrointestinal tract, the presence of a particular plant represents an interaction between the bird and its environment and can have effects on the bird's gut microbial community. In this study, we found that the overwhelmingly important plant taxa in Hawaiian honeycreeper diets were species in the *Metrosideros* genus, ʻōhiʻa. The native ʻōhiʻa is a foundational tree species in Hawaiian rainforests and is central to food webs as a nectar source, home for numerous invertebrates, and as a nesting substrate for birds. However, ʻōhiʻa itself is under threat from Rapid ʻŌhiʻa Death (ROD), which is caused by two different fungal pathogens and has spread rapidly across the Hawaiian archipelago (Barnes et al. 2018; Camp et al. 2019). One of the pathogens, *Ceratocystis lukuohia*, can kill an entire tree within weeks of infection and its presence in Hawaiian forests has already been associated with negative impacts on forest bird populations. Considering the importance of ʻōhiʻa in the diet of honeycreepers and the food web, our results provide further evidence of the importance of protecting this keystone species from ROD.

Hawaiian honeycreepers largely consumed native plant taxa. The only exception was the Oʻahu ʻAmakihi, which mostly ate non‐native plants. Oʻahu is the most urbanized and densely populated island in the archipelago. Almost the entire island has experienced some degree of habitat degradation and plant introductions (Vizentin‐Bugoni et al. 2019), thus it is unsurprising that birds on the island mostly interact with non‐native plants. The introduced Warbling White‐eye appears to similarly interact with both native and non‐native plants. Additionally, this species had the diets with the highest plant richness in the study. The white‐eye's ability to forage in a variety of different substrates aligns with the notion that successful non‐natives can take advantage of a wide range of food resources (Olsson et al. 2009). Because the white‐eye is abundant in a wide variety of habitats across the Hawaiian Islands, assessing their microbiomes and the effects of invertebrate and plant diets both across time and space is a fruitful area for future research.

### 4.2 Honeycreeper Gut Microbiomes

The most abundant bacterial phyla in the gut microbiomes of honeycreepers were Proteobacteria, Firmicutes, and Actinobacteria, which are three of the most typically abundant phyla in avian species (Grond et al. 2018). Bacteroidetes are sometimes also one of the most abundant phyla, but generally only in herbivorous species (Grond et al. 2018; Michel et al. 2018), and this phylum was nearly absent in honeycreeper gut microbiomes. The only species where Bacteroidetes made up at least 10% of the gut microbiome composition was the native granivore, Palila. This species has an interesting natural history, as they are specialists on the māmane plant that has toxic compounds that are extremely poisonous to other bird species (Banko et al. 2002). It is not understood how Palila are able to neutralize the plant‐derived toxins, but their gut microbiomes may play a role in detoxification, as has been documented in other animals (Hansen and Moran 2014; Kohl et al. 2014, 2016). The gut microbiome of the Palila was the second‐most diverse of all species in the study. Considering that they have a specialized diet and a very limited range on Hawaiʻi, this result was unexpected. A more detailed Palila‐specific gut microbiome study is therefore necessary to properly investigate potential mechanisms involved in plant detoxification.

At the phylum level, the relative abundance of particular bacterial taxa remained largely consistent across foraging guilds, islands, and bird species. The bacterial profile of the two introduced species did not appear distinct from native Hawaiian honeycreepers. The only species that appeared different from other species was the Maui ʻAlauahio, which was almost entirely dominated by Firmicutes and had one of the least diverse gut microbiomes in the study. The reason for these differences is not immediately apparent as there was nothing different in the sampling methodology, laboratory work, or bioinformatic analyses, and the samples were collected and processed concurrently with the other species. In addition, neither the invertebrate nor the plant components of its diet were differentiated from other birds based on the COI and ITS2 results. Maui ʻAlauahio is a predominantly insectivorous species, it occupies similar habitats to other native species on Maui, and more detailed diet analyses have not revealed any distinct diet components (Foster, unpublished data). Future studies to investigate its microbiome in more detail are warranted.

### 4.3 Relationship Between Diet Diversity and Microbiome Diversity

In gut microbiome research it is often suggested that there is a positive correlation between diet diversity and gut microbiome diversity and that a reduction in gut microbiome diversity could result in negative health consequences (Heiman and Greenway 2016). We found a significant positive correlation between the invertebrate, but not the plant, diversity of the Hawaiian forest bird diets and their gut microbiome diversity. Birds that ate more different types of invertebrates tended to have more diverse microbiomes. It is possible that a diverse invertebrate diet can introduce new microbes into the gut of an animal, or alternatively (and not mutually exclusive), that a more diverse microbiome may allow for the consumption of a greater variety of prey sources. Because the entire bacterial community of the bird feces is sequenced, there is also the possibility that some of the ASVs are derived from the invertebrates that were consumed (and not actually incorporated into the bird microbiome), however, the same argument could be made for plant‐associated ASVs of which we found no correlation with diversity.

### 4.4 Geography Is a Key Contributor to Hawaiian Honeycreeper Gut Microbiomes

We found little support of phylosymbiosis in the Hawaiian honeycreeper radiation, which is consistent with several other avian studies that have similarly reported weak or no congruence between host phylogeny and gut microbiome composition (Kropačkova et al. 2017; Grond et al. 2019; Loo et al. 2019; Fleischer et al. 2020; Song et al. 2020; Trevelline et al. 2020; Bodawatta et al. 2021).

Instead, sampling island was the only factor that consistently had a significant relationship with the gut microbiome composition of Hawaiian honeycreepers. Host phylogeny repeatedly showed nonsignificant effects on the gut microbiome, with one small exception. While the two phylogenetically informed beta diversity metrics (weighted and unweighted UniFrac) and the nonphylogenetically informed Jaccard metric yielded similar results, the other nonphylogenetically informed metric, Bray–Curtis, showed conflicting patterns regarding host phylogeny. We hypothesize that this difference may be driven by common microbial ASVs being shared in high abundance between closely related species, while rarer ASVs vary by island. In one of the three tests for phylosymbiosis, PaCO, the Bray–Curtis analysis resulted in a significant result. However, for all other beta diversity metrics and tests, birds sampled from the same island, across species and foraging guilds, tended to have more similar microbiomes than birds sampled on other islands. Generally, Kauaʻi samples were distinct in ordination space and mostly formed their own cluster in the species‐by‐island unweighted UniFrac microbial dendrogram, while Maui and Hawaiʻi grouped together. One Oʻahu species clustered with the Kauaʻi samples and one species clustered with the Hawaiʻi/Maui group. This geographic clustering pattern was also displayed in both the plant and invertebrate diet components. These results make sense in the context of the geological timescale of the Hawaiian Islands, which were formed by volcanism in a “conveyor belt” fashion where from east to west the islands get progressively older (Price and Clague 2002). Kauaʻi is the oldest of the main Hawaiian Islands (~5.7 million years) and the farthest geographically from the other main islands (Lerner et al. 2011). In fact, Lerner et al. (2011) suggest that the honeycreeper lineage diversified or radiated into different forms sometime between the formation of Oʻahu and Maui (~4.0–1.9 million years ago). Taken together, it is expected that the bacterial and plant communities of the younger and adjacent Maui and Hawaiʻi are more similar to one another than those of Kauaʻi. These results should be carefully considered in any possible attempts to translocate species between islands, particularly as much of the focus is on moving Kauaʻi endemic species due to their extinction risk from avian malaria. Not only may the prey and vegetative communities differ between islands, but geography also has a role in shaping the host‐associated microbiome of Hawaiian honeycreepers.

### 4.5 Concluding Remarks

Geographical distance was the only variable that showed a consistently significant positive relationship with gut microbiome composition. This relationship may reflect the existence of a unique microbial pool for each Hawaiian island and suggests limited microbial dispersal between islands. Honeycreeper diet was also affected by sampling island. Additionally, a significant positive relationship occurred between the diversity of the invertebrate diet and gut microbiome, meaning that birds that consumed a diverse invertebrate diet had a more diverse gut microbiome. This pattern did not exist for the plant diet. While microbiome composition was species‐specific, we did not find support for phylosymbiosis. Instead, we found that most gut microbiome variation could be attributed to individual differences between birds. This study provides a unique look at how different aspects of the environment affect avian host‐associated gut microbiomes in a recent radiation. Additionally, while most studies that address the link between diet and the gut microbiome in wild animals either utilize a single dietary component (i.e. plants or invertebrates) or only use broad dietary classifications like “foraging guild”, our study incorporates metabarcoding data from both plants and invertebrates. With this knowledge, conservation managers may be able to better care for species *ex situ* (i.e., through dietary or probiotic supplementation in captivity) and in situ (i.e., choosing target areas for translocation).

## Author Contributions

**Maria S. Costantini:** conceptualization (lead), data curation (lead), formal analysis (lead), funding acquisition (lead), investigation (lead), methodology (lead), project administration (equal), resources (equal), software (equal), supervision (lead), validation (lead), visualization (lead), writing – original draft (lead), writing – review and editing (lead). **Elin Videvall:** formal analysis (supporting), methodology (equal), software (equal), visualization (supporting), writing – original draft (supporting), writing – review and editing (equal). **Jeffrey T. Foster:** formal analysis (supporting), methodology (supporting), software (equal), visualization (supporting), writing – review and editing (equal). **Matthew C. I. Medeiros:** conceptualization (supporting), data curation (supporting), formal analysis (supporting), investigation (supporting), methodology (supporting), resources (equal), software (supporting), writing – review and editing (supporting). **John D. Gillece:** formal analysis (supporting), software (equal). **Eben H. Paxton:** resources (equal), writing – review and editing (supporting). **Lisa H. Crampton:** conceptualization (supporting), data curation (supporting), funding acquisition (supporting), project administration (supporting), resources (equal), writing – review and editing (supporting). **Hanna L. Mounce:** resources (equal), writing – review and editing (supporting). **Alex X. Wang:** resources (equal), writing – review and editing (supporting). **Robert C. Fleischer:** conceptualization (supporting), data curation (supporting), funding acquisition (supporting), investigation (supporting), methodology (supporting), resources (equal), software (supporting), writing – original draft (supporting), writing – review and editing (equal). **Michael G. Campana:** data curation (supporting), funding acquisition (supporting), investigation (supporting), methodology (supporting), resources (supporting), supervision (supporting), writing – original draft (supporting), writing – review and editing (equal). **Floyd A. Reed:** conceptualization (equal), data curation (supporting), formal analysis (supporting), funding acquisition (supporting), investigation (supporting), methodology (supporting), resources (equal), software (supporting), writing – original draft (supporting), writing – review and editing (supporting).

## Conflicts of Interest

The authors declare no conflicts of interest.

## Supporting information


**Table S1**.

**Table S2**.

## Data Availability

All sequences are submitted to NCBI under the BioProject number PRJNA1135247. https://dataview.ncbi.nlm.nih.gov/object/PRJNA1135247?reviewer=fq9ojrqp6ch6ehg879o3qpfo83. All code used in analyses is available in a Dryad database at https://datadryad.org/stash/share/3pn1RztOq_a‐JEKCKdrUYelU8LcNXJxoYUGR2JGbeLs.
