# Supplementary material for: The Role of Geography, Diet, and Host Phylogeny on the Gut Microbiome in the Hawaiian Honeycreeper Radiation
Source: Ecol Evol. 2024 Oct 16;14(10):e70372. doi: 10.1002/ece3.70372 (PMC11480636; doi:10.1002/ece3.70372)
Supplement: Supplementary file 1 — Table S1. [file ECE3-14-e70372-s001.docx]

| **Genetic Marker** | **Classified to Phylum** | **Classified to Class** | **Classified to Order** | **Classified to Family** | **Classified to Genus** | **Classified to Species** |
| --- | --- | --- | --- | --- | --- | --- |
| 16S | 99.1% | 99.1% | 98.9% | 97.1% | 86.8% | 58.5% |
| COI | 97.8% | 85.1% | 80.4% | 27.2% | 16.8% | 11.8% |
| ITS2 | 100% | 100% | 100% | 100% | 99.8% | 20.8% |
