# Supplementary material for: The Role of Geography, Diet, and Host Phylogeny on the Gut Microbiome in the Hawaiian Honeycreeper Radiation
Source: Ecol Evol. 2024 Oct 16;14(10):e70372. doi: 10.1002/ece3.70372 (PMC11480636; doi:10.1002/ece3.70372)
Supplement: Supplementary file 2 — Table S2. [file ECE3-14-e70372-s002.docx]

| **Table S2. MRM test results (permutations = 9999) for all possible model combinations predicting the full microbiome across they honeycreeper phylogeny. Significant values are bolded.** | | | | | | | | | |
| --- | --- | --- | --- | --- | --- | --- | --- | --- | --- |
|  |  |  |  |  |  |  |  |  |  |
| **Model** | **Number of parameters** | **Statistic** | **Plant** | **Invert** | **Geographic distance** | **Phylogeny** | **Model R^2^** | **Model F** | **Model p** |
| 1 | 1 | F | -0.02 |  |  |  | 0.0002 | 0.04 | 0.87 |
|  |  | p | 0.87 |  |  |  |  |  |  |
| 2 | 1 | F |  | -0.04 |  |  | 0.0026 | 0.50 | 0.64 |
|  |  | p |  | 0.64 |  |  |  |  |  |
| **3** | **1** | **F** |  |  | **0.02** |  | **0.1665** | **37.56** | **<0.01** |
|  |  | **p** |  |  | **<0.01** |  |  |  |  |
| 4 | 1 | F |  |  |  | 0.15 | 0.0454 | 8.94 | 0.09 |
|  |  | p |  |  |  | 0.09 |  |  |  |
| **5** | **2** | **F** |  |  | **0.02** | **0.17** | **0.2248** | **27.11** | **<0.01** |
|  |  | **p** |  |  | **<0.01** | **0.03** |  |  |  |
| **6** | **2** | **F** |  | -0.04 | **0.02** |  | **0.1686** | **18.96** | **<0.01** |
|  |  | **p** |  | 0.66 | **<0.01** |  |  |  |  |
| 7 | 2 | F |  | -0.10 |  | 0.17 | 0.0574 | 5.69 | 0.11 |
|  |  | p |  | 0.30 |  | 0.05 |  |  |  |
| **8** | **2** | **F** | 0.04 |  | **0.02** |  | **0.1681** | **18.89** | **<0.01** |
|  |  | **p** | 0.64 |  | **<0.01** |  |  |  |  |
| 9 | 2 | F | -0.03 |  |  | 0.15 | 0.0461 | 4.52 | 0.15 |
|  |  | p | 0.77 |  |  | 0.09 |  |  |  |
| 10 | 2 | F | 0.02 | -0.05 |  |  | 0.0028 | 0.26 | 0.87 |
|  |  | p | 0.88 | 0.64 |  |  |  |  |  |
| **11** | **3** | **F** | 0.03 |  | **0.02** | **0.17** | **0.2256** | **18.06** | **<0.01** |
|  |  | **p** | 0.73 |  | **<0.01** | **0.03** |  |  |  |
| **12** | **3** | **F** |  | -0.10 | **0.02** | **0.19** | **0.2370** | **19.26** | **<0.01** |
|  |  | **p** |  | 0.23 | **<0.01** | **0.01** |  |  |  |
| 13 | 3 | F | 0.05 | -0.12 |  | 0.17 | 0.0588 | 3.87 | 0.18 |
|  |  | p | 0.69 | 0.29 |  | 0.05 |  |  |  |
| **14** | **3** | **F** | 0.09 | -0.08 | **0.02** |  | **0.1743** | **13.01** | **<0.01** |
|  |  | **p** | 0.37 | 0.44 | **<0.01** |  |  |  |  |
| **15** | **4** | **F** | 0.13 | -0.16 | **0.02** | **0.20** | **0.2480** | **15.25** | **<0.01** |
|  |  | **p** | 0.19 | 0.11 | **<0.01** | **0.01** |  |  |  |
